# A**_3_** Adenosine Receptor Allosteric Modulator Induces an Anti-Inflammatory Effect: *In Vivo* Studies and Molecular Mechanism of Action

**DOI:** 10.1155/2014/708746

**Published:** 2014-10-13

**Authors:** Shira Cohen, Faina Barer, Sara Bar-Yehuda, Adriaan P. IJzerman, Kenneth A. Jacobson, Pnina Fishman

**Affiliations:** ^1^Can-Fite BioPharma Ltd., Kiryat-Matalon, 10 Bareket Street, P.O. Box 7537, 49170 Petach Tikva, Israel; ^2^Division of Medicinal Chemistry, Leiden Academic Centre for Drug Research, Leiden University, P.O. Box 9502, 2300 RA Leiden, The Netherlands; ^3^Molecular Recognition Section, Laboratory of Bioorganic Chemistry, National Institute of Diabetes and Digestive and Kidney Diseases, National Institutes of Health, Bethesda, MD 20892-0810, USA

## Abstract

The A_3_ adenosine receptor (A_3_AR) is overexpressed in inflammatory cells and in the peripheral blood mononuclear cells of individuals with inflammatory conditions. Agonists to the A_3_AR are known to induce specific anti-inflammatory effects upon chronic treatment. LUF6000 is an allosteric compound known to modulate the A_3_AR and render the endogenous ligand adenosine to bind to the receptor with higher affinity. The advantage of allosteric modulators is their capability to target specifically areas where adenosine levels are increased such as inflammatory and tumor sites, whereas normal body cells and tissues are refractory to the allosteric modulators due to low adenosine levels. LUF6000 administration induced anti-inflammatory effect in 3 experimental animal models of rat adjuvant induced arthritis, monoiodoacetate induced osteoarthritis, and concanavalin A induced liver inflammation in mice. The molecular mechanism of action points to deregulation of signaling proteins including PI3K, IKK, I*κ*B, Jak-2, and STAT-1, resulting in decreased levels of NF-*κ*B, known to mediate inflammatory effects. Moreover, LUF6000 induced a slight stimulatory effect on the number of normal white blood cells and neutrophils. The anti-inflammatory effect of LUF6000, mechanism of action, and the differential effects on inflammatory and normal cells position this allosteric modulator as an attractive and unique drug candidate.

## 1. Introduction

The A_3_ adenosine receptor (A_3_AR) belongs to the family of the Gi-protein coupled receptors (GPCR). Much evidence including preclinical and clinical studies has accumulated showing anti-inflammatory effects that are mediated via the A_3_AR [1–8]. A_3_AR agonists induce anti-inflammatory effects in both murine and rat models of autoimmune arthritis [2, 9], via downregulation of nuclear factor kappa B (NF-*κ*B) and related proteins as well as tumor necrosis factor-*α* (TNF*α*) [2], resulting in the inhibition of inflammatory cytokines [1, 10, 11]. A_3_AR is overexpressed in cells from inflammatory tissues whereas normal cells have a low expression of the receptor [12, 13]. Furthermore, A_3_AR was found to be upregulated in the peripheral blood mononuclear cells (PBMCs) of patients with autoimmune inflammatory diseases [13–16]. PBMCs drawn from rheumatoid arthritis (RA), psoriasis, and Crohn's disease (CD) patients showed A_3_AR upregulation compared to that of healthy subjects, suggesting that the high A_3_AR expression levels in the inflammatory tissues are reflected in the PBMCs [15].

A_3_AR agonists such as CF101 (IB-MECA) and CF102 (Cl-IB-MECA) have been investigated in several phase II clinical studies including RA, psoriasis, and hepatocellular carcinoma, showing clear evidence of efficacy and an excellent safety profile [17–19], proof of the validity of the A_3_AR as a therapeutic target.

While A_3_AR agonists bind at an orthosteric receptor binding site to induce activation, a positive allosteric modulator (PAM) would bind to an additional binding site on the receptor that is distinct from the agonist binding site, thereby enhancing the affinity and/or efficacy of the natural ligand adenosine at the A_3_AR. The advantage of allosteric modulators is their capability to target specifically areas where adenosine levels are increased such as at inflammatory and tumor sites, whereas normal body cells and tissues are in theory refractory to the allosteric modulators due to lower adenosine levels [20].

**Figure 1:**
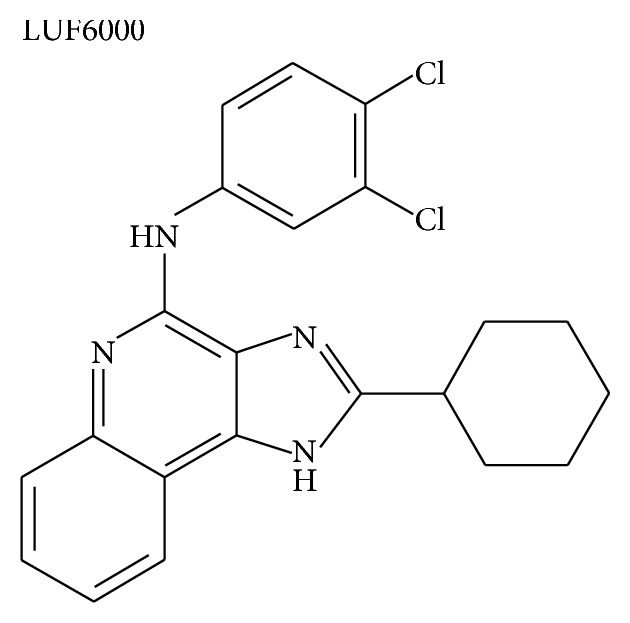
Chemical structure of the A_3_AR allosteric enhancer, LUF6000, used in this study.

N-(3,4-Dichloro-phenyl)-2-cyclohexyl-lH-imidazo [4,5-c]quinolin-4-amine (LUF6000) is an imidazoquinolinamine allosteric enhancer of the human A_3_AR that upon binding changes the native ligand interaction with the receptor and raises its maximal effect by 45% [21] (Figure 1).

In a [^35^S]GTP*γ*S binding assay, LUF6000 was able to convert the nucleoside antagonist MRS542 into an A_3_AR agonist and was found to be highly effective in raising the maximal effect of low-efficacy agonists. LUF6000 alone did not induce receptor activation and therefore is potentially safer than orthosteric agonists [22].

In this study we present data showing that LUF6000 has a specific and potent anti-inflammatory effect* in vivo*. A molecular mechanism associated with these effects is presented as well.

## 2. Materials and Methods

### 2.1. Reagents

The allosteric modulator LUF6000 (N-(3,4-dichloro-phenyl)-2-cyclohexyl-lH-imidazo [4,5-c]quinolin-4-amine) was synthesized for Can-Fite BioPharma at Leiden Academic Centre for Drug Research (Leiden, The Netherlands)/Haoyuan Chemexpress Co., Ltd (Shanghai, China). A stock solution of 10 mM was prepared in DMSO and further dilutions were prepared in PBS. ConA (Canavalia ensiformis, Jack Bean Hemagglutinin) was purchased from Calbiochem-EMD Millipore (San Diego, CA). Monosodium iodoacetate (MIA; Sigma, St. Louis, MO) was prepared in saline solution.

Rabbit polyclonal antibodies against rat A_3_AR, phosphoinositide 3-kinase (PI3K), I*κ*B kinase (IKK), I*κ*B, nuclear factor kappa B (NF-*κ*B), Janus kinase 2 (Jak-2), and signal transducer and activator transcription 1 (STAT-1), glyceraldehyde-3-phosphate dehydrogenase (GAPDH), and *β*-actin were purchased from Santa Cruz Biotechnology, Inc. (Dallas, TX, USA).

### 2.2. Effect of LUF6000 on the Development of Adjuvant Induced Arthritis

Female Lewis rats, 9 weeks of age, were obtained from Harlan Laboratories (Jerusalem, Israel). Rats were maintained on a standardized pelleted diet and supplied with tap water. Experiments were performed in accordance with the guidelines established by the Institutional Animal Care and Use Committee at Can-Fite BioPharma, Petach Tikva, Israel. The rats were injected subcutaneously (SC) at the tail base with 100 *μ*L of suspension composed of incomplete Freund's adjuvant (IFA) with 10 mg/mL heat killed* Mycobacterium tuberculosis*, (Mt) H37Ra, (Difco, Detroit, USA). Each group contained 10 animals. LUF6000 (100 *μ*g/kg) treatment was orally administered by gavage, thrice daily, starting with the disease onset. The control group received vehicle only (DMSO at a dilution corresponding to that of the DMSO stock solution of LUF6000).

A clinical disease activity score was assessed, blinded, as follows: the animals were inspected every day for clinical signs of arthritis. The scoring system ranged from 0 to 4 of each limb: 0, no arthritis; 1, redness or swelling of one toe/finger joint; 2, redness and swelling of more than one toe/finger joints, 3, the ankle and tarsal-metatarsal joints involvement; 4, redness or swelling of the entire paw. The clinical score was calculated by adding the four individual legs' score to a maximum.

At the end of the study, rats were sacrificed using a CO_2_ method. The hind paws were dissected above the ankle joint. In addition, blood samples were collected and subjected to a Ficoll-hypaque gradient. The PBMCs were then washed with PBS and protein extracts were prepared as is detailed below.

The bony tissue was broken into pieces, snap frozen in liquid nitrogen, and stored at −80°C until use. The paw tissues were added to (4 mL/g tissue) radioimmunoprecipitation assay (RIPA) extraction buffer containing 150 mM NaCl, 50 mM Tris, 1% NP40, 0.5% deoxycholate, and 0.1% SDS. Tissues were homogenized on ice with a polytron, centrifuged and the supernatants were subjected to Western Blot analysis. Each group included 10 animals, and the study was repeated at least 3 times.

### 2.3. MIA Induced OA Experimental Model

Male Wistar rats (150–175 g) were obtained from Harlan Laboratories (Jerusalem, Israel). Rats were maintained on a standardized pelleted diet and supplied with tap water. Experiments were performed in accordance with the guidelines established by the Institutional Animal Care and Use Committee at Can-Fite BioPharma, Petach Tikva, Israel.

OA experimental model was induced with MIA, 2 mg at a total volume of 50 *μ*L. The MIA was injected intra-articularly through the patellar ligament of the right knee using a 26 G needle under anesthesia. The left knee joint (control) was injected with saline.

Oral treatment with LUF6000, 100 *μ*g/kg, BID started on day 7 (after MIA injection) and lasted until the termination of the study. The control group was treated with the LUF6000 vehicle. Each group included 10 animals, and the study was repeated at least 3 times.

The diameter of the knees was measured every other day using a digital caliper (Mitotoyo, Tokyo, Japan).

### 2.4. Liver Inflammation Model

Male C57BL/6J mice 8 weeks of age were obtained from Harlan Laboratories (Jerusalem, Israel). The animals were maintained on a standardized pelleted diet and supplied with tap water. Experiments were performed in accordance with the guidelines established by the Institutional Animal Care and Use Committee at Can-Fite BioPharma, Petach-Tikva, Israel.

Male C57BL/6J mice were injected intravenously (tail vein) with concanavalin A (Con A) (20 mg/kg). LUF6000 was orally administered by gavage, in a dose of 10 and 100 *μ*g/kg, twice daily starting 8 h after Con A administration. The control group received only DMSO in a dilution corresponding to a 100 *μ*g/kg dose of LUF6000. Blood samples were collected 21 h after Con A administration from the retroorbital vein and serum levels of liver enzymes (SGOT and SGPT) were determined. Mice were sacrificed by CO_2_ inhalation method. The livers were subjected to Western blot and pathological analysis. Each group included 8–10 mice, and the study was repeated 3 times.

### 2.5. Differential Blood Cell Count upon Oral Treatment with LUF6000

ICR male mice (23–25 g) were treated thrice daily with LUF6000, 100 *μ*g/kg for 48 hours. Blood samples were withdrawn 24 and 48 hours after the last treatment. A differential blood cell count was performed. Each group included 10 mice, and the study was repeated 4 times.

### 2.6. Western Blot Analysis of A_3_AR and Additional Signaling Proteins in PBMCs

Western blot analyses were carried out according to the following protocol. Samples were rinsed with ice-cold PBS and transferred to ice-cold lysis buffer (TNN buffer, 50 mM Tris buffer pH = 7.5, 150 mM NaCl, NP 40). Cell debris was removed by centrifugation for 10 min, at 7500 ×g. Protein concentrations were determined using the Bio-Rad protein assay dye reagent. Equal amounts of the sample (50 *μ*g) were separated by SDS-PAGE, using 12% polyacrylamide gels. The resolved proteins were then electroblotted onto nitrocellulose membranes (Schleicher & Schuell, Keene, NH, USA). Membranes were blocked with 1% BSA and incubated with the desired primary antibody (dilution 1 : 1000) for 24 h at 4°C. Blots were then washed and incubated with a secondary antibody for 1 h at room temperature. Bands were recorded using BCIP/NBT color development kit (Promega, Madison, W1, USA). Units were determined by calculation of the ratio between the housekeeping gene and the subjected protein. *β*-actin was used in the MIA and AIA models, while GAPDH was used in the liver inflammation model due to previous experiments indicating a much accurate results with the GAPDH in the liver.

### 2.7. Statistical Analysis

The results were evaluated using Student's *t*-test, with statistical significance set at *P* < 0.05. Comparison between the mean values of different experiments was carried out. All data are reported as mean ± SD.

## 3. Results

### 3.1. LUF6000 Inhibits the Development of Adjuvant Induced Arthritis (AIA)

**Figure 2:**
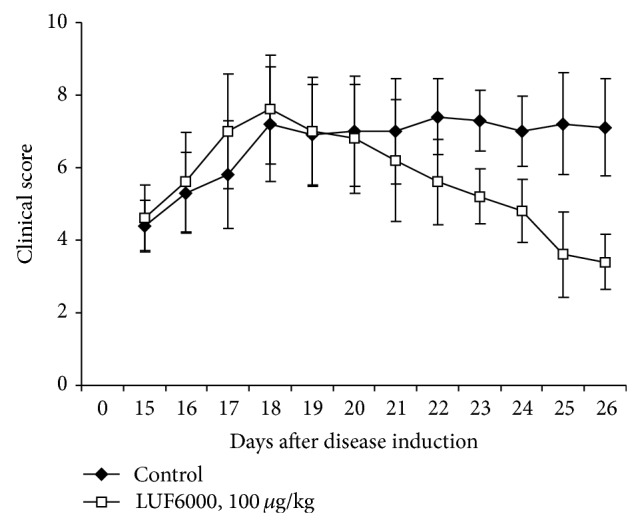
Effect of LUF6000 on the clinical manifestation of adjuvant induced arthritis (AIA) in a rat model of RA. Lewis rats were injected subcutaneously (SC) at the tail base with complete Freund's adjuvant. LUF6000 (100 *μ*g/kg) treatment was given PO, thrice daily, starting upon disease onset. LUF6000 decreased disease manifestation compared to the vehicle-treated group. Values are the mean of pooled data from 3 independent experiments (*n* = 10).

LUF6000 reduced the RA clinical score in an adjuvant induced arthritis rat model, demonstrating a significant anti-inflammatory effect (Figure 2).

**Figure 3:**
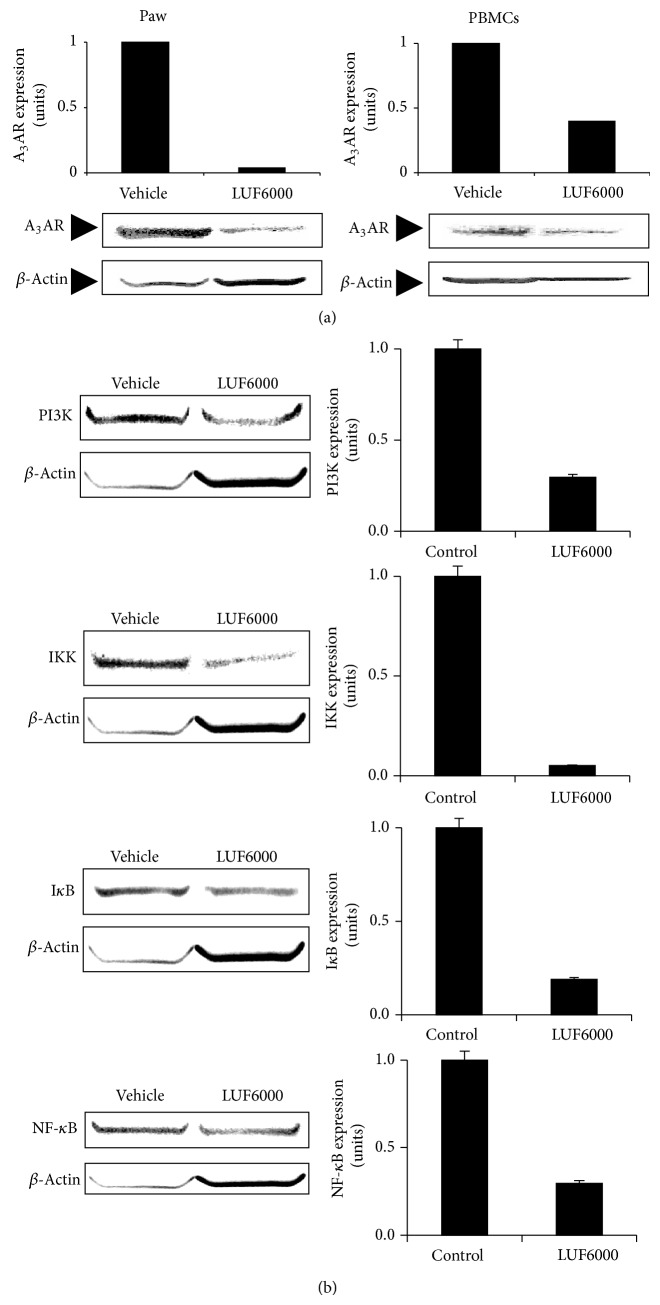
LUF6000 downregulates the NF-*κ*B signaling pathway via the A_3_AR. Protein analysis was performed using Western blot (WB) analysis in both PAW and PBMCs. LUF6000 downregulates A_3_AR expression levels in both PAW and PBMCs (a). PI3K, IKK, I*κ*B, and NF-*κ*B, all members of the NF-*κ*B signaling pathway, were downregulated upon LUF6000 administration (b).

A_3_AR expression levels in paw extracts and in the PBMCs were downregulated in the LUF6000-treated rats compared to the vehicle-treated ones (Figure 3(a)), demonstrating that A_3_AR levels in the PBMCs are a reflection of the receptor level in the remote inflammatory organ. PI3K, IKK and I*κ*B expression levels in the PBMCs were downregulated upon treatment with LUF6000 resulting in a decrease in NF-*κ*B expression levels (Figure 3(b)).

### 3.2. LUF6000 Inhibits the Development of MIA-Induced Osteoarthritis (OA)

**Figure 4:**
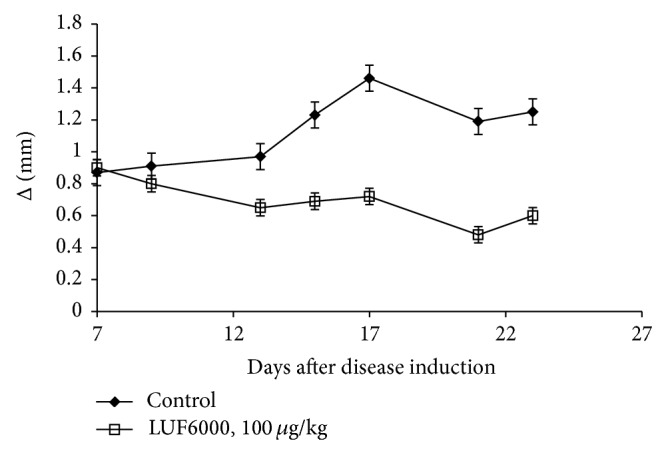
LUF6000 effect on disease manifestation in a monosodium iodoacetate (MIA) model of osteoarthritis (OA) in rats. The OA experimental model was induced by injection of MIA to the intra-articularly through the patellar ligament of the right knee. LUF6000 (100 *μ*g/kg) administered PO; BID starting upon onset of disease showed a decrease in disease manifestation compared to the vehicle-treated group. Values are the mean of pooled data from 3 independent experiments (*n* = 10).

**Figure 5:**
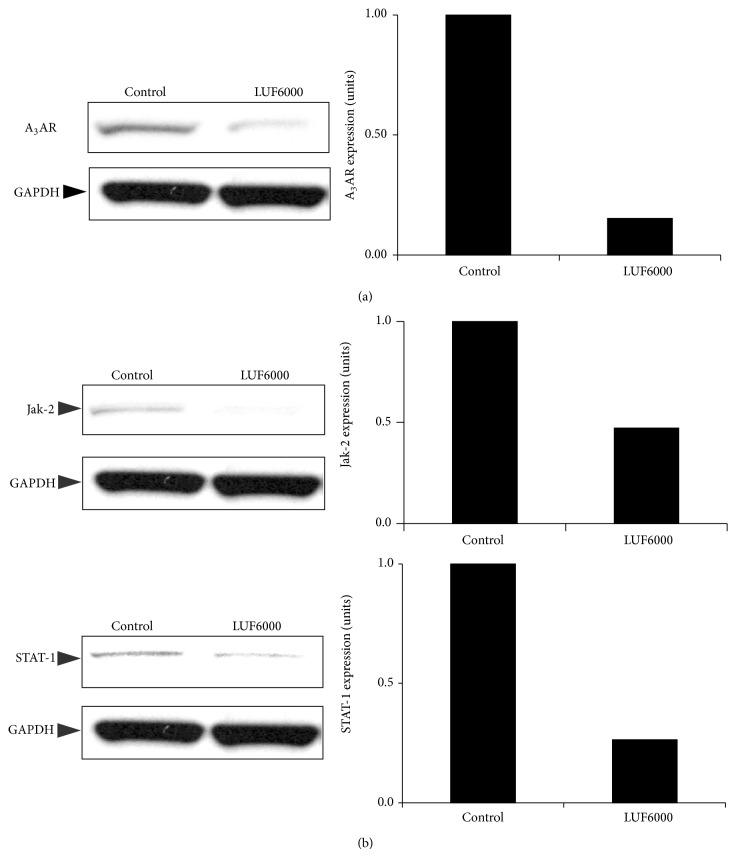
LUF6000 down-regulates the A_3_AR and key inflammatory proteins. Protein analysis was performed using WB analysis. LUF6000 downregulates A_3_AR expression levels in the PBMCs of OA-induced rats (a). Jak-2 and STAT-1 were downregulated upon LUF6000 administration (b).

LUF6000 inhibited osteoarthritis development in an experimental rat model manifested by a decrease in knee swelling and edema in the LUF6000-treated group compared to the vehicle (Figure 4). A_3_AR expression levels in the PBMCs were down regulated in the LUF6000-treated group (Figure 5(a)) followed by down regulation of the expression levels of the inflammatory proteins Jak-2 and STAT-1 compared to the vehicle-treated group (Figure 5(b)).

### 3.3. LUF6000 Protects against Liver Inflammation

**Figure 6:**
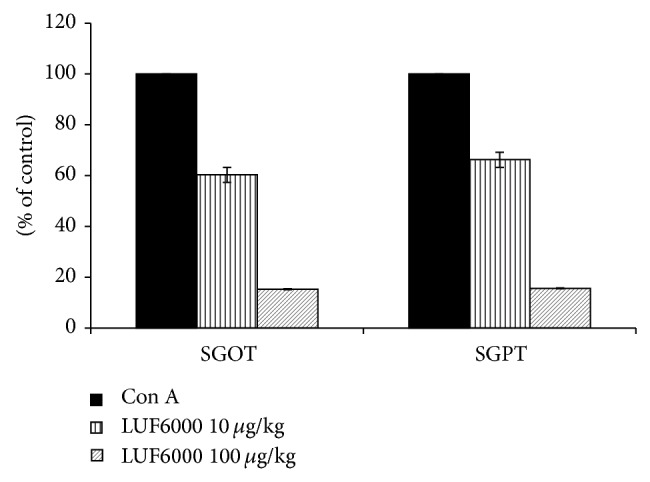
Effect of LUF6000 on the development of a Con A-induced hepatitis model in mice. LUF6000 (10 and 100 *μ*g/kg), administered twice daily PO, starting upon the disease onset, markedly decreased both SGOT and SGPT liver enzymes levels compared to the vehicle in a dose-dependent manner. Values are the mean of pooled data from 3 independent experiments (*n* = 10).

The A_3_AR agonist, CF102, had been found to have a protective effect in a liver inflammation model of acute hepatitis in mice [23]. In the current study we explored the effect of LUF6000 on liver inflammation induced by Con A induction. A decrease was observed in serum glutamic pyruvate transaminase (SGPT) and serum glutamic oxaloacetic transaminase (SGOT) levels, compared to the vehicle-treated group (Figure 6). LUF6000 showed a protective effect in both 10 and 100 *μ*g/kg groups, resulting in dose-dependency with the 100 *μ*g/kg dose demonstrating a better protective effect than at 10 *μ*g/kg.

### 3.4. LUF6000 Induces an Increase in WBCs and Neutrophils

**Figure 7:**
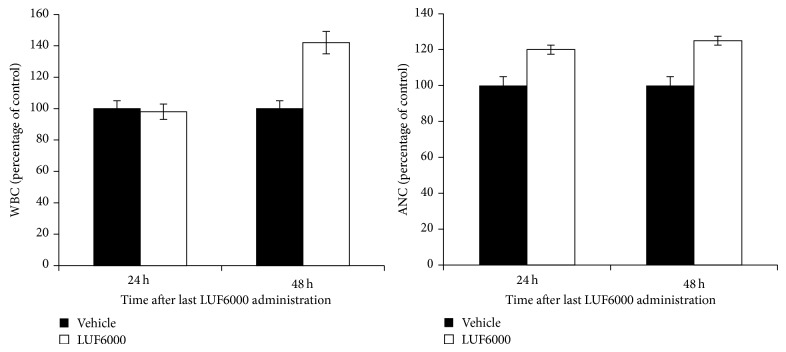
LUF6000 induces an increase in white blood cells total count and neutrophils (ANC). LUF6000 (100 *μ*g/kg), was administered, PO, thrice daily for 48 hours to ICR male mice. Blood samples were withdrawn 24 and 48 h after the last treatment. LUF6000 increased levels of both WBCs and neutrophils. Values are the mean of pooled data from 3 independent experiments (*n* = 10).

LUF6000 administration to normal ICR mice for 2 sequential days resulted in an increase in white blood cell (WBC) count at 48 h after the last treatment. Neutrophil counts were increased at both 24 h and 48 h after LUF6000 administration demonstrating a slight stimulatory effect on bone marrow myeloid cells (Figure 7).

## 4. Discussion

Small molecules that bind to topographically distinct sites on GPCRs are known to generate a conformational change in the orthosteric site of the receptor, thereby modulating the affinity or efficacy of natural ligands. Such allosteric modulators, which may alternately enhance or reduce the effect of the native agonist, are considered as safe and efficacious drug candidates.

LUF6000 is an imidazoquinolinamine allosteric modulator at the human A_3_AR, known to enhance the efficacy of receptor agonists acting at the receptor's orthosteric site. This was shown in both [^35^S]GTP*γ*S binding assays [22] and a number of other functional assays [23]. Accumulation of the endogenous agonist adenosine in the microenvironment of inflammatory sites has been extensively documented and serves as a good basis to utilize LUF6000 as an allosteric ligand of A_3_AR to evoke a specific anti-inflammatory effect. LUF6000 was also shown to enhance the agonist effect of inosine, which serves as a second, albeit weak, endogenous agonist at the human A_3_AR [21–23].

In this study we have looked at the effect of the allosteric modulator LUF6000 on three different experimental animal models, sharing common mechanistic pathways.

In the AIA and MIA models, an antiarthritic effect, manifested by a reduced clinical score of the disease, was observed. Interestingly A_3_AR expression levels in the animals' PBMCs were found to be downregulated, demonstrating that receptor modulation, most probably internalization and degradation took place. The downstream molecular mechanism entailed inhibition in the expression of PI3K, IKK, and I*κ*B resulting in inhibition of NF-*κ*B. This mechanistic pathway corroborates with the one described for A_3_AR agonists, supporting the notion that the natural ligand adenosine mediated the response and that LUF6000 is a positive allosteric modulator, other mechanisms cannot be excluded though, and it should be kept in mind that A_3_AR ligand pharmacology may differ between rodent (in the present study) and man [22, 23].

This study is the first to present data showing the anti-inflammatory effect of LUF6000* in vivo*. The role of A_3_AR in mediating inflammatory responses has been extensively described in preclinical and clinical studies. A_3_AR upregulation has been described in patients with RA [24] and in animals in which arthritis was induced [2]. Receptor upregulation has been attributed to transcription factors such as NF-*κ*B, known to be overexpressed in arthritis. Treatment with A_3_AR agonists such as CF101 and CF502 led to a marked improvement in disease parameters* in vivo* [2, 4, 25]. In experimental animal models, downregulation of the NF-*κ*B and the Wnt signaling pathways has been shown to mediate the anti-inflammatory effect of the A_3_AR agonists [2, 25]. The mechanistic pathway described in this study shows that LUF6000 induces its anti-inflammatory effect via a similar mechanistic pathway as described earlier for A_3_AR agonists.

An additional mechanism explored in the OA model entailed a decrease in the inflammatory proteins STAT-1 and Jak-2. Inhibition of Jak-2 is known to block STAT-1 activation as well as matrix metalloproteinase 13 in chondrocytes, resulting in the protection of chondrocytes and cartilage in OA [26].

We next checked the effect of LUF6000 in a liver inflammation model of Con A-induced hepatitis in mice. LUF6000 administration resulted in a decrease of liver enzymes, thus counteracting the effect of Con A in a dose-dependent manner. These results indicate that LUF6000 administration has a protective effect on the liver. Earlier studies showed that A_3_AR agonists have a hepatoprotective effect on the liver mediated via downregulation of TNF*α* levels and inhibition of the apoptotic proteins Bax and Bad [26, 27].

The effect of LUF6000 on normal cells was explored upon its administration to ICR mice and the followup on peripheral WBC and neutrophil counts. Interestingly, WBC counts were normal upon treatment with LUF6000, and even a slight increase in neutrophil numbers was noted. This effect demonstrated that LUF6000 has differential effects on normal and pathological cells, again, similar to the effect of the A_3_AR agonists IB-MECA and Cl-IB-MECA, which were mediated via the secretion of granulocyte colony-stimulating factor (G-CSF) [27–32].

## 5. Conclusion

LUF6000 has been shown to be effective when given orally, inducing specific anti-inflammatory effects, rendering this molecule to be considered as a potential drug candidate.
